# Introducing ATR-FTIR Spectroscopy through Analysis of Acetaminophen Drugs: Practical Lessons for Interdisciplinary and Progressive Learning for Undergraduate Students

**DOI:** 10.1021/acs.jchemed.0c01231

**Published:** 2021-07-12

**Authors:** Félix Zapata, Adrián López-Fernández, Fernando Ortega-Ojeda, Gloria Quintanilla, Carmen García-Ruiz, Gemma Montalvo

**Affiliations:** ^†^Department of Analytical Chemistry, Physical Chemistry, and Chemical Engineering, ^‡^Department of Physics and Mathematics, ^§^University Institute of Research in Police Sciences (IUICP), and ^∥^Department of Organic Chemistry and Inorganic Chemistry, University of Alcalá, Ctra. Madrid-Barcelona km 33.6, 28871 Alcalá de Henares, Madrid, Spain

**Keywords:** Upper-Division Undergraduate, Undergraduate Research, Analytical Chemistry, Qualitative Analysis, Forensic Chemistry, Hands-On Learning/Manipulatives, Laboratory Equipment/Apparatus, Drugs/Pharmaceuticals, IR Spectroscopy, Chemometrics

## Abstract

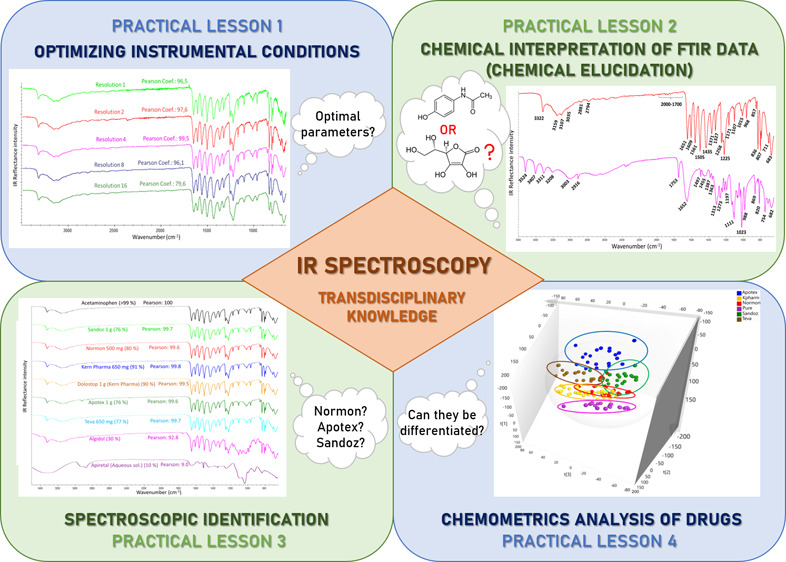

Infrared (IR) spectroscopy is a vibrational spectroscopic technique useful in chemical, pharmaceutical, and forensic sciences. It is essential to identify chemicals for reasons spanning from scientific research and academic practices to quality control in companies. However, in some university degrees, graduate students do not get the proficiency to optimize the experimental parameters to obtain the best IR spectra; to correlate the IR spectral bands with the molecular vibrations (chemical elucidation); to have some criteria for any substance identification (especially relevant in quality control to recognize counterfeit); and to apply chemometrics for comparing, visualizing, and classifying the IR spectra. This work presents an experimental laboratory practice for an introductory teaching of the IR instrumental conditions in the identification of substances based on visual spectra comparison and statistical analysis and matching. Then, the selected IR conditions are applied to different commercial drugs, in the solid state or in solution, mostly composed of acetaminophen. Finally, the students apply chemometrics analysis to the IR data. This practice was designed for the training in a chemistry subject for undergraduate students of the chemistry, pharmacy, or forensics degrees, among others related to science, medical, food, or technological sciences.

## Introduction

IR spectroscopy is a vibrational spectroscopic technique available in most laboratories to identify chemical substances; it is useful from scientific research and academic practices to quality control in companies.^1,2−3^ It is usually the technique that is first employed for identification purposes because it is nondestructive, rapid, selective, and solvent-free. In addition, when using the attenuated total reflectance (ATR) mode, sample treatment is not needed, and thus, the sample remains intact after the spectroscopic analysis in such a way that can be kept for further analyses. Beyond experimental facilities, a comprehensive use of the instrumentation is mandatory for a correct interpretation of the spectral results. The aim of this work is to present an experimental laboratory practice composed of different modules for teaching and training: (1) the influence of instrumental conditions, such as resolution and number of scans, (2) the chemical interpretation of spectral bands, (3) the identification of substances based on visual spectra comparison and database matching, and (4) the discrimination of samples using chemometry. To introduce students to the topic, several commercial drugs are studied whose main active pharmaceutical ingredient (API) is paracetamol (i.e., acetaminophen), but that differ in their brands and/or dose as well as in the pharmaceutical form. The different experiments are collected and presented for the training of the university undergraduate students taking chemical subjects in the pharmacy, chemistry, and forensics degrees, among other scientific disciplines.

## Pedagogical Aims

In the organic and inorganic chemistry subjects, the teaching of Fourier transform infrared spectroscopy (FTIR) is very common. On one hand, in the theoretical lessons of physical/organic chemistry in most universities, the students usually learn the correlation between the different molecular vibrations and their corresponding absorption wavelengths. On the other hand, in the laboratory practices in the analytical/organic/inorganic chemistry subjects, the students obtain the spectra and check the characteristic vibrational bands of a synthesized compound. However, in many universities, their training in IR spectroscopy is neither complete nor integrated, mainly because of different absences in their curricula. They do not know how to (a) optimize the experimental parameters to get the best spectra, (b) experimentally correlate IR spectral bands with molecular vibrations (chemical elucidation), (c) have some criteria for substance identification which is especially relevant in quality control to recognize drug adulteration, and (d) apply multivariate analysis to discriminate among similar samples.

Before tackling these experiments, the students are advised to take some introductory lessons on FTIR spectroscopy, IR vibrational interpretation, and statistics. To assist the teachers in selecting and compiling the appropriate content for these lessons, a very fundamental introduction to these topics can be found in the Supporting Information. In this way, the students’ interest and commitment would increase, making them predisposed to a more significant, meaningful, and practical learning. This article proposes modular, collaborative, and progressive learning of attenuated total reflection FTIR (ATR-FTIR) spectroscopy for the analysis and discrimination of acetaminophen drugs. This learning is based on the following questions:

(1) How to select the optimal instrumental conditions?
(2) How to undertake the chemical elucidation using the FTIR data?
(3) How to identify a compound with high confidence using spectral libraries?
(4) How to discriminate among samples with similar IR spectra using chemometrics?

The analysis of pharmaceutical drugs to teach and train students in using IR spectroscopy is not novel, and several examples can be found in the literature.^4−7^ However, most of them are exclusively focused on the practical training of the ATR-FTIR technique and/or the vibrational chemical interpretation of the IR spectra.^8^ However, the curricula of some chemistry or pharmacy university degrees may not include any training on the comparison of spectral libraries or the spectral matching using, for instance, the Pearson coefficient. Likewise, they may not include the subsequent use of chemometrics to improve the discrimination of samples based on the compounds’ specific IR spectral features. The sequential modular experimental practice presented in this article seeks to cover these currently relevant aspects when teaching and training university undergraduate students in the ATR-FTIR spectroscopy field. Higher education goes toward more transdisciplinary degree proposals; thus, the collaborative teaching among the subjects results in relevant, consistent, and integrated learning. It should be indicated that, for the moment, the students did not participate on any specific tests to quantitatively evaluate their learning level as a consequence of conducting these experiments. The qualitative assessment, to date, is only based on the students’ supervision performed during their laboratory work through the in situ verification of their progressive tasks.

## Experimental Section

### Materials

The ascorbic acid and acetaminophen standards (>99%) were purchased from MilliporeSigma (aka Sigma-Aldrich, Merck KGaA, St. Louis, MI, USA). All the eight drugs commercially available in Spain had paracetamol (acetaminophen) as their main active pharmaceutical ingredient (API). All of those compounds were analyzed by undergraduate students. The students determined that the tablets and the oral solution were visually and microscopically homogeneous, whereas the powder for oral solution, composed of three main APIs, was heterogeneous even to the naked eye. The students were also asked to weigh the eight drugs to experimentally determine their acetaminophen composition. The reference they used was the API dose indicated by the manufacturer. Table 1 summarizes the name, brand, pharmaceutical form, dose, and main composition of each drug. This table was filled in by the students grouped in pairs.

**Table 1:** Synopsis of the Commercial Drugs Containing Acetaminophen Analyzed in This Experiment

				Main Composition^a^		
Drug Name	Brand Name	Pharmaceutical form	Dose	API^b^	API,^b^ %	Excipients, %
Paracetamol	Sandoz	Tablets	1 g	Acetaminophen	76	24
Paracetamol	Apotex	Tablets	1 g	Acetaminophen	76	24
Dolostop	Kern Pharma	Tablets	1 g	Acetaminophen	90	10
Paracetamol	Kern Pharma	Tablets	650 mg	Acetaminophen	91	9
Paracetamol	Teva	Tablets	650 mg	Acetaminophen	77	23
Paracetamol	Normon	Tablets	500 mg	Acetaminophen	80	20
Apiretal	ERN S.A.	Oral solution	100 mg/mL	Acetaminophen	10	90^c^
Algidol	Almirall S.A.	Powder for oral solution	650 mg	Acetaminophen	30	46.5
				Ascorbic acid	23	
				Codeine	0.5	

a Experimentally calculated by weighing the tablet in an analytical balance (two replicates) and assuming the dose (indicated by the manufacturer) as the real amount of acetaminophen. The main composition was calculated as follows: % acetaminophen = (dose/weighted mass) × 100; % excipients = 100 – % acetaminophen – % other active pharmaceutical ingredients.

b API, active pharmaceutical ingredient.

c The oral solution includes water with the excipients.

### Instrumentation

All weighing was carried out using an Ohaus DV215CD analytical balance (Parsippany, NJ, USA) with a precision of five decimal places (i.e., 0.00001 g).

The IR analyses were performed with an FTIR Nicolet IS10 spectrometer (Thermo Scientific, Waltham, MA, USA) equipped with a smart ITR module for ATR measurements in the spectral range from 3500 to 650 cm^–1^ and operated with the Omnic 9 software for IR spectroscopy (Thermo Scientific, Waltham, MA, USA).

### Procedure

The laboratory practice presented herein is composed of four modules/lessons, with a 4 h per day pace. Lessons 1, 2, and 3 were carried out in the chemistry laboratory, while the fourth lesson was carried out in a computer room.

The students paired work, allowed them to surpass the individual mind-scheme, reinforcing the discussion in pairs which, in turn, cemented the development of their argumentation, synthesis, critical judgment, and communication skills. In addition, strengthening the concepts understanding further improves the students’ self-confidence.

The procedure for the different proposed lessons was as follows:

1. In lesson 1, the paired students were asked to analyze 0.1–0.2 g (about a tip of spatula) of acetaminophen and ascorbic acid pure standards (>99%) by ATR-FTIR spectroscopy using different instrumental conditions.

- Effect of the resolution. The students were asked to compare the ATR-FTIR spectra of the acetaminophen standard recorded at different values of resolution, 1, 2, 4, 8, and 16 cm^–1^, while keeping constant the spectral range (3500–650 cm^–1^) and the number of scans (16).
- Effect of the number of scans. The students were also asked to compare the ATR-FTIR spectra of the acetaminophen standard recorded by accumulating different number of scans: 1, 4, 8, 16, and 32, while keeping constant the spectral range (3500–650 cm^–1^) and a resolution of 4 cm^–1^.

2. In lesson 2, the students were requested to analyze the acetaminophen and ascorbic acid standards by ATR-FTIR spectroscopy using the best conditions set found in the previous lesson (resolution (4 cm^–1^) and number of scans (16)). The students included those spectra into a homemade spectral library and discussed (in pairs) the vibrational interpretation of the spectral bands.
3. In lesson 3, the students were asked to analyze the eight pharmaceutical forms mentioned above by ATR-FTIR spectroscopy using the best conditions set found in lesson 1. Tablets do not make a good contact with the ATR diamond crystal; thus, the students should realize that they need to powder them down in a mortar. Then, the students placed about 0.1–0.2 g (a tip of spatula) onto the ATR crystal and pressed the powder making sure the powder covered the crystal completely. The FTIR spectrum was then recorded using the optimum conditions (spectral range from 3500 to 650 cm^–1^, resolution of 4 cm^–1^, and 16 number of scans). Between replicates, the students were asked to stir the powder on the ATR crystal and press it again. This way, a new contact sample-ATR occurred for each replicate which is a recommended procedure in the case of mixtures and heterogeneous samples. The students were requested to analyze two different tablets of each brand-dose and collect, at least, 10 replicates for each tablet. Therefore, each pair of students should have collected a total of 20 FTIR spectra for each formulation. The students were then asked to visually and automatically compare the spectra of commercial drugs to the spectrum of acetaminophen standard previously included in the library. The corresponding Pearson coefficient value for the statistical matching can be obtained using the built-in function in the Omnic software.
4. In lesson 4, the students were questioned about the possibility of discriminating the different drugs using chemometrics. Prior to any analysis, the raw spectra underwent a series of common cleansing spectral procedures to make them comparable while getting most of the available information. Those mathematical procedures were offset and baseline correction, normalization (standard normal variates, SNV), and smoothing (seven-points Savitzky–Golay).^9^ The spectra were preprocessed using the free R (v4.0)^10^ within RStudio (v1.3)^11^ software. The Statgraphics Centurion 18 software (Statgraphics Tecnologies, Virginia, USA) was used since it is powerful, is friendly enough for novices, and offers various advise for helping the students better interpret the analysis outcomes. The SIMCA 15 software (Sartorius Stedim Biotech, Göttingen, Germany) was also used because this multivariate analysis software is very powerful and rather easy to use while tuning the different plotting options for understanding the samples’ behavior. The data was previously centered and scaled (unit vector), and the software was set to calculate the boundaries with 95% probability to counterweigh for any magnitude unbalance and/or variance that might exist.^12^ This procedure allowed eliminating any weight contribution due to the variables or observations magnitude. For eluding any model overfitting, the final number of components was based on the autofitting cross-validation setting as suggested by the principal component analysis (PCA) module in SIMCA.

## Hazards

All samples are marketable pharmaceuticals in Spain. Particularly, they are nontoxic, and no safety equipment (laboratory coat, safety gloves, and glasses) is really needed, though they might be worn as usual when working in a laboratory.

## Results and Discussion

Besides training the specific competences of each practical lesson discussed below, the joint achievement of the four lessons will provide a progressive interdisciplinary and integrative learning of IR spectroscopy and chemometrics to students, hardly obtainable separately.

### Practical Lesson 1. Optimizing Instrumental Conditions

First of all, it is important for the students to become familiar with the influence of the instrumental parameters on the IR spectrum. To visualize the effect, two laboratory experiments were proposed: (1) optimizing the resolution and (2) optimizing the number of scans. In these experiments, the students’ aim was to make decisions on whether the spectrum quality can be improved by optimizing the instrumental conditions. The students had to check the different influences of the various instrumental parameters on the quality of the FTIR spectrum.

#### Resolution

**Figure 1:**
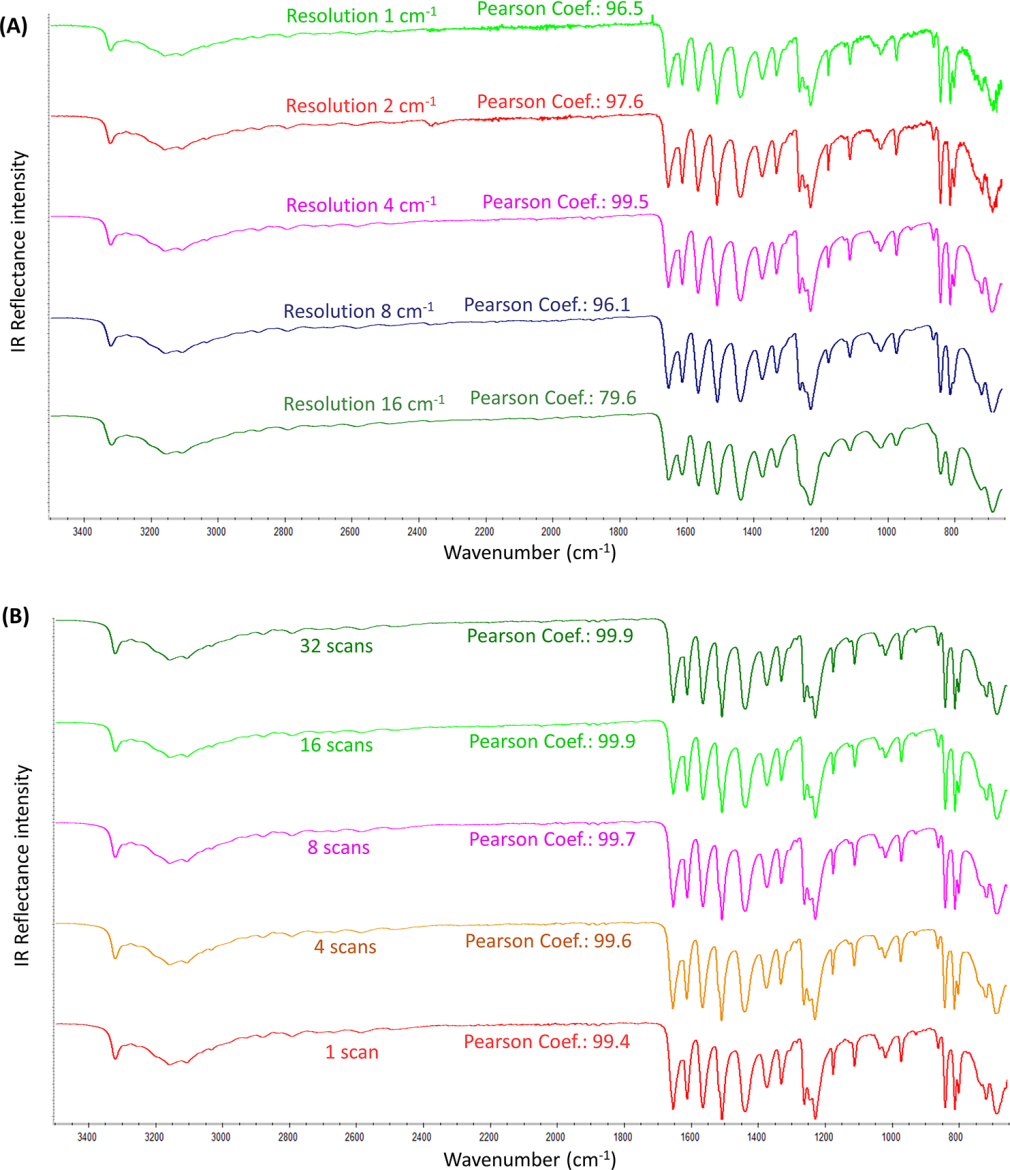
(A) ATR-FTIR spectra of the acetaminophen standard (>99%) collected at different values of resolution, while keeping constant the spectral range (3500–650 cm^–1^) and the number of scans (16). (B) ATR-FTIR spectra of the acetaminophen standard (>99%) collecting different numbers of scans, while keeping constant the spectral range (3500–650 cm^–1^) and resolution (4 cm^–1^). The corresponding Pearson coefficient of each spectrum versus the acetaminophen standard spectrum displayed in Figure 2 (reference spectrum) is given.

The resolution strongly influences the definition of the IR spectrum, i.e., the sharpness of the IR signals, which ultimately depends on the data spacing. The smaller the data spacing is, the smaller the resolution value is, the better the resolution is, and the higher the definition of the IR spectrum is. The students observed this by comparing the 16 cm^–1^ resolution spectrum (poor resolution) with the other resolution values (Figure 1A). The IR bands at 16 cm^–1^ resolution are not correctly defined, even to the point that some bands totally disappear because of an overlapping with closer, more intense, bands. If only the sharpness of the IR spectrum was considered, the resolution of 1 would be the best. However, the spectral noise greatly increased when using very high resolution (especially 1 and 2 cm^–1^). Altogether, by comparing the IR spectra of Figure 1A, and considering both factors (sharpness and noise), a resolution of 4 cm^–1^ might be selected as the optimum resolution since it provided the best ratio of highest definition and least spectral noise.

#### Number of Scans

The number of scans influenced the spectral noise. Since the IR spectrum is the mathematical average of all collected scans, the higher the number of scans is, the lesser the instrumental noise is, and thus, the better the signal-to-noise ratio is. Nonetheless, the spectral noise differences within the spectra (Figure 1B) were hardly detectable to the students’ naked eye. Positively, the statistical analysis of the spectra through the Pearson correlation (used as supporting data) provided evidence that the same Pearson value was obtained for the 16 and 32 scans. Since time matters, 16 scans might be selected as the optimum number of scans.

### Practical Lesson 2. Chemical Interpretation of FTIR Data (Chemical Elucidation)

**Figure 2:**
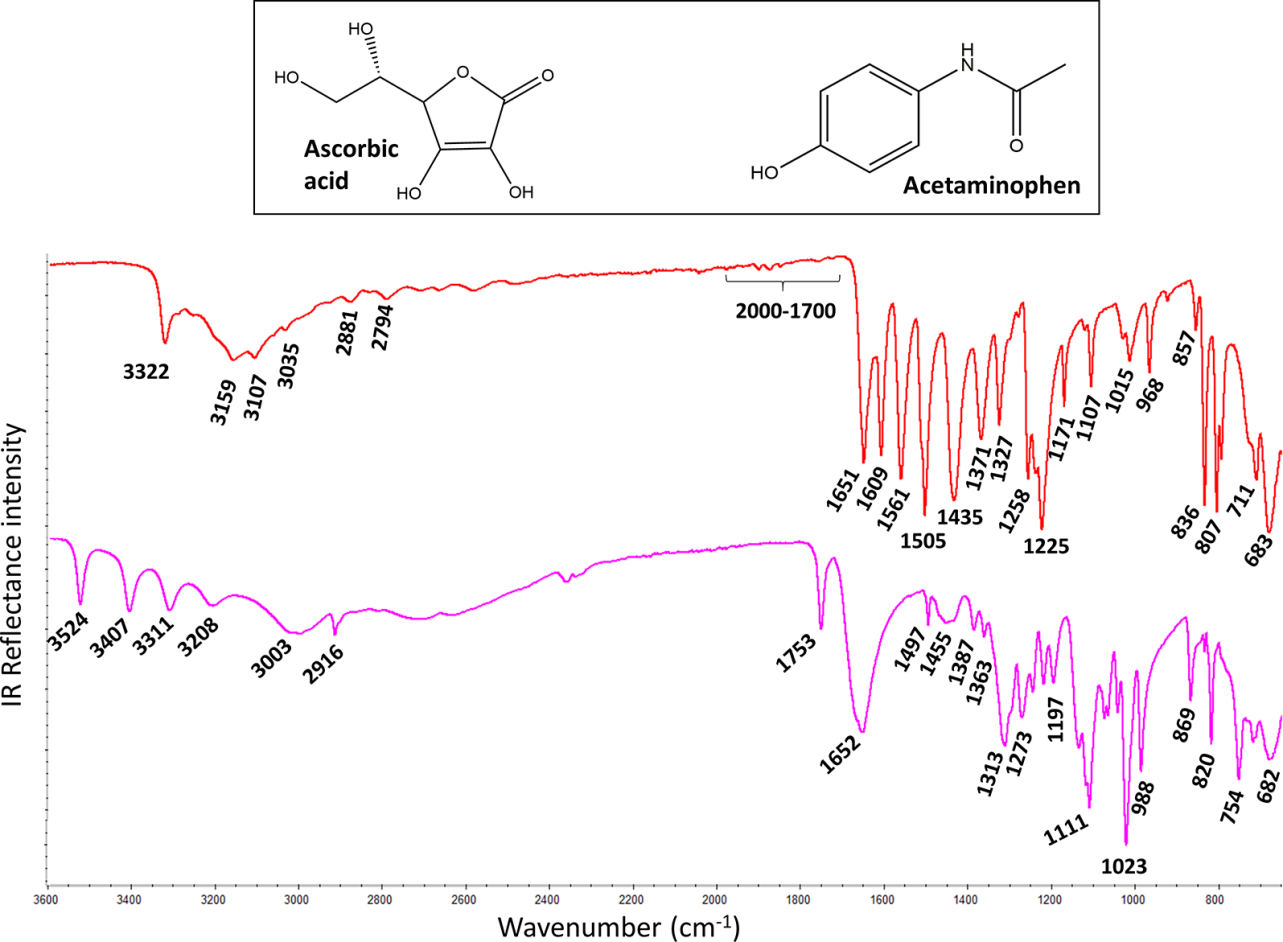
Average ATR-FTIR spectra of two-sample problem. FTIR conditions: number of scans (16) and resolution (4 cm^–1^). The real samples are the acetaminophen (red, above) and ascorbic acid (purple, below) standards (>99%). The students know neither the molecular formula nor their corresponding spectrum assignments until the resolution of the problem.

Within the second practical lesson, the student is expected to acquire the competence to chemically interpret the IR spectra. In order to awaken the students’ interest, the teacher proposed a challenge to the students: “*Let’s suppose for example that some students forgot to put the name when collecting the FTIR spectra of the ascorbic acid (vitamin C) and acetaminophen (paracetamol) standards*. *Both spectra are now displayed in the computer screen (*Figure 2*)*, *but the students are not sure which is which*. *Could anyone know and reason which spectrum belongs to each compound*?”

The IR absorption bands are due to molecular vibrations. Furthermore, the comprehensive interpretation and assignment of each band with the corresponding molecular vibration is possible due to the combined study of the empirical IR spectra obtained over the years for different molecular structures and the development of quantum mechanics. In fact, for spectral-structure interpretation purposes, specific narrow ranges have been established for each molecular vibration. University students must understand and know the meaning of the spectral bands and identify to what type of chemical bonds they could be attributed. Infrared spectral interpretation for chemical elucidation is an important lesson in organic chemistry courses. In the literature, there are multiple interpretation tables listing some spectroscopy wavenumber ranges and the appearance of the vibration bands for different functional groups.^13−16^

In this experiment, the students used the tables from the IR chapter in Pretsch et al.,^13^ which is the handbook normally used in theoretical lessons. Through this practice, the students exercised how to use the IR interpretation tables in order to match correctly the IR spectrum with its corresponding compound (acetaminophen or ascorbic acid). First, the students compared the chemical structures of both acetaminophen and ascorbic acid, identifying the chemical vibrations, i.e., chemical bonds, which are different for both compounds. Second, the students searched in the IR handbook for the wavenumber ranges at which the IR bands appear due to the stretching and bending vibrations of those bonds. Third, the students looked for and found those IR bands in their experimental spectra (Figure 2) in order to match each experimental spectrum with the corresponding compound.

In brief, the acetaminophen molecule has the following bonds: O–H (phenol), N–H (amide), C(sp^2^)–H (aromatic), C(sp^3^)–H (methyl group), C=O (amide), C=C (aromatic), C–O (alcohol), and C–N–C (amide). On the other hand, the ascorbic acid has the following bonds: O–H (four alcohol groups), C(sp^3^)–H (CH or CH_2_ groups), C=O (cyclic ester (lactone)), C=C (double bond), C–O (alcohol), and C–O–C (lactone). Thus, the main structural differences of acetaminophen and ascorbic acid involve the presence of N–H (amide), C(sp^2^)–H (aromatic), and C–N (amide) in acetaminophen versus C–O–C (cyclic ester) in ascorbic acid. Additional differences involve that (i) C=O belongs to an amide group in acetaminophen, but to a cyclic ester in ascorbic acid; (ii) C=C is aromatic in acetaminophen, but a double bond in ascorbic acid; (iii) C(sp^3^)–H belongs to a methyl group in acetaminophen, but to CH and CH_2_ in ascorbic acid; and (iv) ascorbic acid has four alcohol groups while acetaminophen has only one phenolic alcohol. At this point, it might be worthwhile for the students to note that ascorbic acid, despite its name, is not a carboxylic acid but a cyclic ester, which may assist the teacher in doing related experiments comparing both chemical groups. Though those studies are out of the scope of this lesson, they serve as an example to show the interest and relevance of the interrelation in learning.

Afterward, the students searched in the interpretation table for the wavenumber ranges within which the molecular vibrations of previous chemical bonds/groups usually absorb. The teacher helped the students by indicating the molecular vibrations occurring in the acetaminophen and ascorbic acid molecules (bold in Table 2). As a result, the students completed Table 2 by indicating the corresponding wavenumber ranges found in the literature for the fundamental molecular vibrations of acetaminophen and ascorbic acid. The teacher gave the students a reasonable time frame (from one session/day to the next) to prepare this table. Then, it was discussed and corrected as a team until the full information was completed.

**Table 2:**
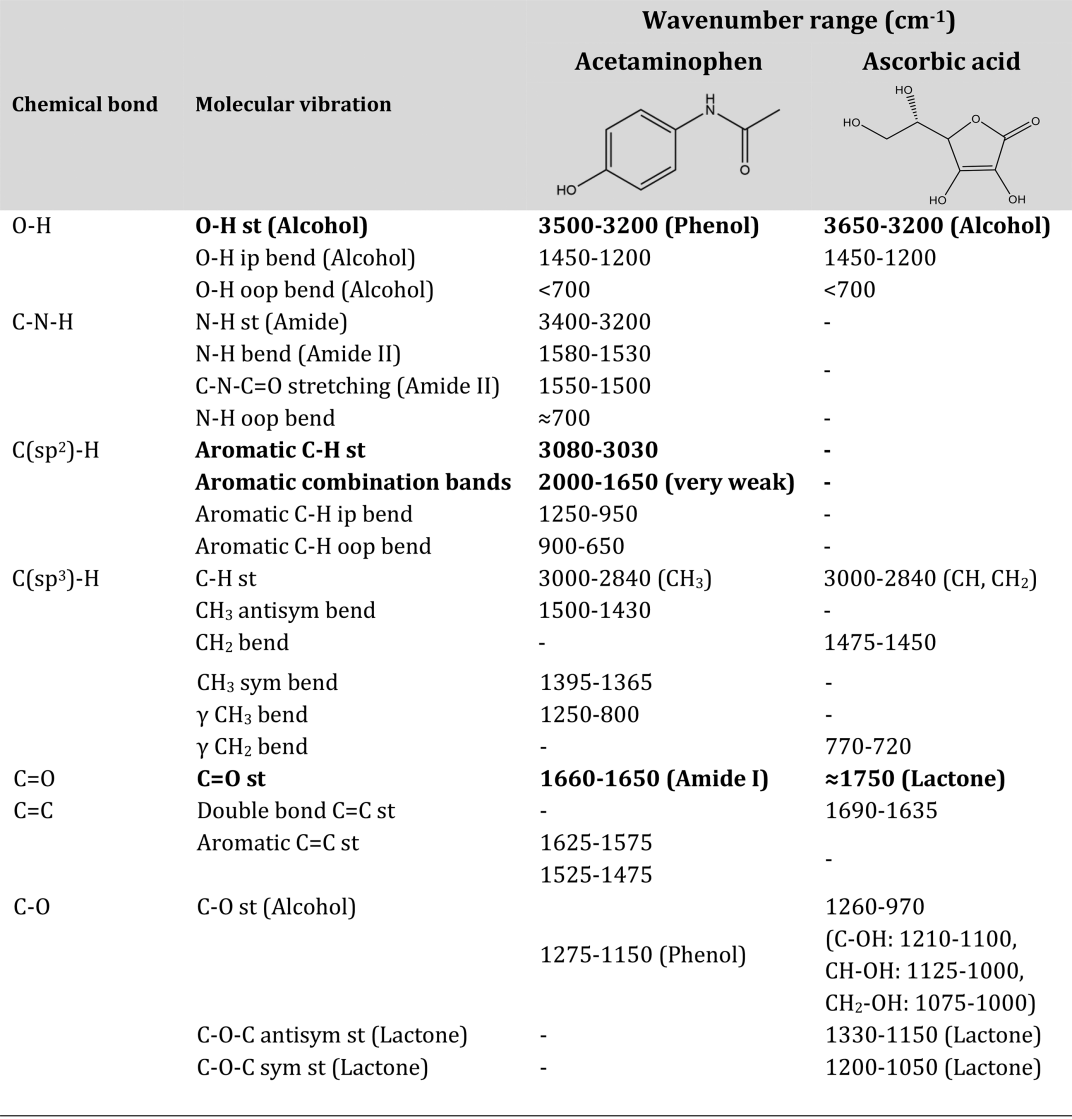
Summary of the Fundamental Molecular Vibrations Expected for Acetaminophen and Ascorbic Acid, and Their Corresponding Wavenumber Range According to IR Literature^a^

a See ref (13).

As summarized in Table 2, a major spectral difference between acetaminophen and ascorbic acid is the C=O stretching between an amide and a lactone (1650 vs 1750 cm^–1^). This fact is highly relevant because the C=O stretching provides a very intense IR band that locates within a wavenumber range (1800–1650 cm^–1^) in which normally there are no overlapping bands from other molecular vibrations. In this case, by comparing the IR spectra (Figure 2) in the search for C=O stretching, it is easy to identify the C=O band due to a lactone (1753 cm^–1^) in the purple IR spectrum. Therefore, this finding would be enough to confirm that the purple spectrum belongs to ascorbic acid rather than to acetaminophen. Moreover, additional findings support this identification. For example, the red spectrum displays (a) very weak bands between 2000 and 1700 cm^–1^, which are the combination bands characteristic for aromatics; and (b) the band at 3035 cm^–1^ which is due to C(sp^2^)–H, which is only possible for acetaminophen. In addition, the presence of several O–H groups (ascorbic acid) is clearly observed in the purple spectrum because there are multiple bands between 3600 and 3200 cm^–1^, which are due to the O–H stretching. However, the O–H stretching from phenol and the N–H stretching from amide (acetaminophen) are observed in the red spectrum. To finish the study, the students were encouraged to find, in the IR spectra (Figure 2), the remaining molecular vibrations summarized in Table 2. It should be noticed that all IR bands experimentally observed in Figure 2 for acetaminophen and ascorbic acid were in accordance with the data previously reported in the literature.^16−18^ The skilled students were encouraged to compare their reasoned band-vibration assignments with the detailed assessment to fundamental molecular vibrations reported in advanced spectroscopic–computational studies^17−19^ for these two molecules.

### Practical Lesson 3. Spectroscopic Identification

The following lesson was prepared with the objective for the students to acquire the ability to use IR spectral libraries for identifying compounds with a high statistical confidence. In order to identify an “unknown” substance, the sample IR spectrum is usually compared with spectral databases containing the spectra of numerous standard substances. A significant number of standards’ spectra are usually included by default in the various spectrometer software programs. This number can be increased by either purchasing additional spectral libraries from spectroscopic instrumentation companies for different prices and/or developing homemade spectral libraries by including new standard substances analyzed in the laboratory over time. The sample spectrum vs spectral database comparison performed by the software normally provides a list of potential candidates together with a decreasing numerical matching. In most spectroscopic software, this numerical matching ranging from 0% to 100% is usually calculated through the Pearson correlation. This is a simple statistical analysis that measures the correlation, i.e., similarity, of two variables. The more similar the spectra are, the higher the matching is. For instance, the matching between two spectra that are completely identical will be 100%.

The software usually lists the substances exclusively according to their statistical matching value. However, the visual comparison of the potential candidates spectra must be accomplished in order to ensure a positive identification. The presence or absence of characteristic spectral bands strongly supports the positive or negative identification of the compound of interest among the different candidates automatically listed.

**Figure 3:**
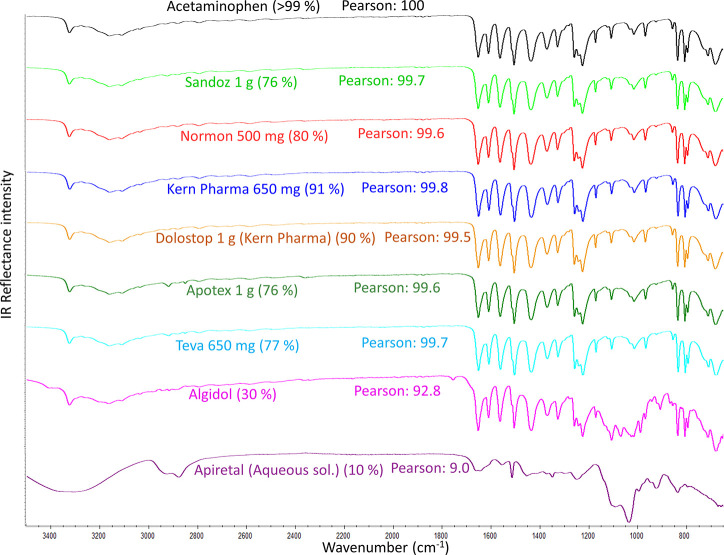
Average ATR-FTIR spectra of the acetaminophen standard (>99%) and commercial drugs. The spectrum of each drug is the average of 20 spectra collected from two EFG tablets. IR conditions: spectral range (3500–650 cm^–1^), resolution (4 cm^–1^), and number of scans (16) for each of the 20 spectra. The corresponding Pearson coefficient of each drug versus the acetaminophen standard (reference spectrum) is given.

In this practice, the compound of interest is acetaminophen. Particularly, the aim is to identify acetaminophen in different pharmaceutical formulations. In this respect, the students were asked to automatically compare the spectrum of each drug formulation against the spectrum of the acetaminophen standard previously included in the library. The results are shown in Figure 3. The IR spectra of the drugs mostly composed of acetaminophen (>75%) matched almost exactly the spectrum of the acetaminophen standard. Their Pearson correlation exceeded 99.5%. Only Algidol (a drug composed of 30% acetaminophen) and Apiretal (an aqueous solution composed of 10% acetaminophen) provided lower Pearson coefficient values when compared to the acetaminophen standard. Nevertheless, despite being only composed of 30% acetaminophen, Algidol clearly displayed the characteristic bands of acetaminophen and highly matched the spectrum of the acetaminophen standard (92.8%). On the contrary, the characteristic bands of acetaminophen were not detected in the spectrum of the Apiretal aqueous solution. Hence, its Pearson matching against the acetaminophen standard was minimal (9%). As expected, the Apiretal IR spectrum was dominated by highly IR-active water bands, which overlapped any possible signal of acetaminophen.

As previously discussed, the FTIR spectra correlation for the different acetaminophen solid presentations was mostly similar to the pure standard, being difficult to differentiate among the drugs. This similarity does not necessarily mean that all the pharmaceutical presentations have the same excipients, but that the excipients’ bands hardly contribute to the drug spectrum, which are almost exclusively due to the acetaminophen. This way, the students learned that this technique will not allow them to identify separate signals from the excipients and APIs, but there is instead a global FTIR spectrum of the sample. In addition, it is important to note that the Pearson % is related to the spectral numerical coincidences and does not indicate percentages in composition or purity in the mixtures.

### Practical Lesson 4. Chemometric Analysis of the Drugs to Differentiate the Brands or Dose

In various science fields, chemistry and statistics are taught simultaneously or in progressive courses. As mentioned before, the collaboration among the different courses in the same degree can increase relevant learning. On one hand, the mathematical data treatment can also give a chemical meaning because the IR spectra represent the chemical fingerprint of the analyzed compounds. On the other hand, in many research areas, more than one IR spectrum is often collected for each sample. Therefore, the use of a bunch of IR spectra for studying chemometrics can be an opportunity to make it relevant while reaching significant learning in both subjects. This chemometrics lesson gives the students evidence that the use of multivariate statistics is an extraordinary supporting tool to tackle the limited discrimination of the IR spectra when using only a visual comparison and/or the Pearson correlation matching. In essence, this fourth lesson proposes a clear need for connecting the chemistry, spectroscopy, and statistics courses in the undergraduate curriculum.

Although the IR spectrum is usually dominated by the signal of the main sample component (i.e., the API in the case of medicines), the IR spectrum is the sum of the spectral signals given by all the medicinal product components according to their amount (ratio) and their IR activity. Thus, different formulations based on the same API usually result in similar spectra but likely with slight differences. While such differences are sometimes evident to the naked eye, they are always detectable when using chemometrics.^20−26^ In other words, chemometrics enables the detection of those subtle disparities, allowing the discrimination of similar samples like drugs from different brands or doses, which have slightly different compositions because of varying the excipients or their ratios. For this fourth lesson, the students were taken from the chemistry laboratory to a computer room, in which each student (one computer per student) followed and reproduced the chemometric analyses performed by the teacher in her/his computer (streamed to the class). It should be noted that, the previous day, the students copied the IR spectra from the IR instrument’s computer to a virtual accessible location. Hence, the students analyzed their own IR spectra.

Before starting with the IR spectra chemometric analysis, the students were first instructed to check if the studied data samples were parametric (normally distributed) or nonparametric (non-normally distributed), if their variances were equal (homoscedastic) or nonequal (heteroscedastic), if they were dependent (related) or independent (nonrelated), etc. In this respect, the IR spectra were non-normally distributed while exhibiting nonequality in their variance. This is important to know to select the most appropriate chemometric test. In this case, independent nonparametric more-than-two samples would be analyzed using the Kruskal–Wallis test whereas independent parametric more-than-two samples with equal variances would be analyzed using the ANOVA procedure.

Although there are many chemometric methods (Figure S2), the proposed course includes some basic chemometric concepts and methods for comparing (i), visualizing (ii), and classifying (iii) the IR spectra.

#### i Comparison

**Figure 4:**
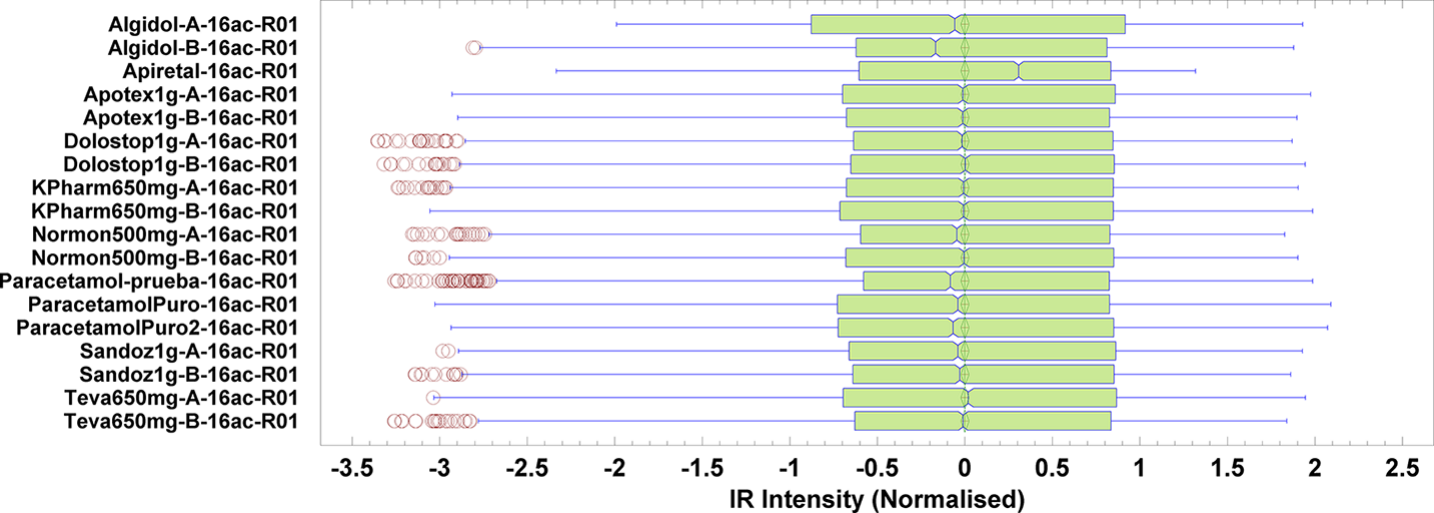
Box-and-whisker plot for the 18 randomly selected spectra studied in this work.

In this spectral context, comparison involves examining whether multiple samples come from the same source. For the nonparametric IR spectra, the types of nonparametric analysis techniques useful for comparing the samples are the Kruskal–Wallis, Jonckheer, and Friedman tests.^26^ In this particular data set, the data comparison was done using the Kruskal–Wallis multiple sample comparison test, which is suitable to compare small sets of samples, especially if few variables are involved in the analysis. This test analyzes the variance; specifically, it checks if there is a difference in the median values of three or more independent samples.^27^ This test is similar to the Mann–Whitney test which ranks the original data values. The Kruskal–Wallis test allows a comparison of many spectra (columns, in the Statgraphics software) at the same time (menu in the software: Compare/Multiple Samples/Multiple-Samples Comparison).^28^ One could compare samples (like drugs) from the same batch, type, brand, dose, etc., or samples totally unrelated to each other. In this lesson, one random sample (spectrum) was selected from every kind of sample spectra; that is, the multiple sample comparison was performed on a set composed of 18 nonrelated, nonparametric, and heteroscedastic samples (spectra). Basically, most statistic studies must begin with a descriptive statistics stage used as exploratory data analysis. This is useful to understand and illustrate the important features of the data matrix, the variables, their distribution and range, and the possible presence of outliers (abnormal data), etc. According to this type of data, several descriptive tables and graphs can be selected on the appropriate software window. The students were asked to focus on the box-and-whisker plot (boxplot, for short), which summarizes the sample using five statistics (minimum, quartile, median, upper quartile, and maximum), and it may also indicate the presence of outliers. In this example, some spectra (e.g., Algidol-B-16ac-R01, Apiretal-16ac-R01, and Paracetamol-prueba-16ac-R01) were more different from others; their lower and upper limits and medians were quite deviated (Figure 4). The standardized skewness and kurtosis were outside the (−2 to +2) range for the 18 spectra, which indicates some significant non-normality in the data. The students were also requested to perform the Kruskal–Wallis test, which checks the null hypothesis that the medians within each of the 18 columns are the same; that is, it assesses whether there are any significant differences among the spectra medians. In the case of these IR spectra, since the resulting *p*-value was 0.026 (less than the 0.05 significance level), there was a statistically significant difference among the spectra medians at the 95.0% confidence level.

#### ii Visualization

**Figure 5:**
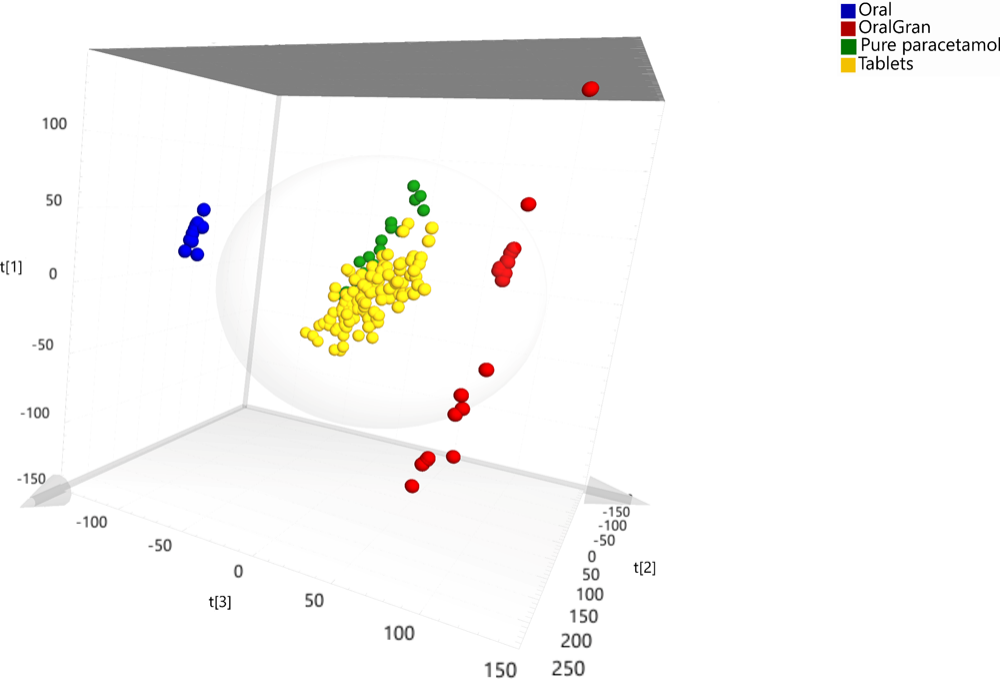
PCA scores scatter 3D plot for all the spectra samples in the data set. The samples are colored by the Pharmaceutical form. Oral and oral gran are clearly out of the Hotelling’s T2 95% bubble.

Visualization is a fairly simple concept pursuing an illustration of the data and/or the statistics calculated in a chart. Among the chemometric visualization approaches, PCA is the most widely used as a data (variables) exploration, a reduction method, and because ideally it allows the data visualization and differentiation of large sample matrices containing many observations and variables at the same time. The students were asked to calculate the PCA model, as it was performed by the teacher on the streaming. As an example, Figure 5 shows the PCA score scatter 3D plot for all the spectra in one of the students’ data sets. Here, the samples are colored depending on their pharmaceutical form, i.e., solid tablets, solid powder, or liquid solution. It is important to notice that the samples located out of the Hotelling’s T2 95% bubble were exceptionally statistically different samples with respect to the samples placed inside the bubble. Hence, they would represent statistically outlier samples in this PCA model.

As the students expected, such different spectra outside the bubble were the spectra from the Algidol powder for oral solution and from the Apiretal liquid solution. Thus, PCA allowed to easily differentiate the various intake forms (i.e., oral liquid solution, powder for oral solution, and tablets, and powdered pure acetaminophen).

**Figure 6:**
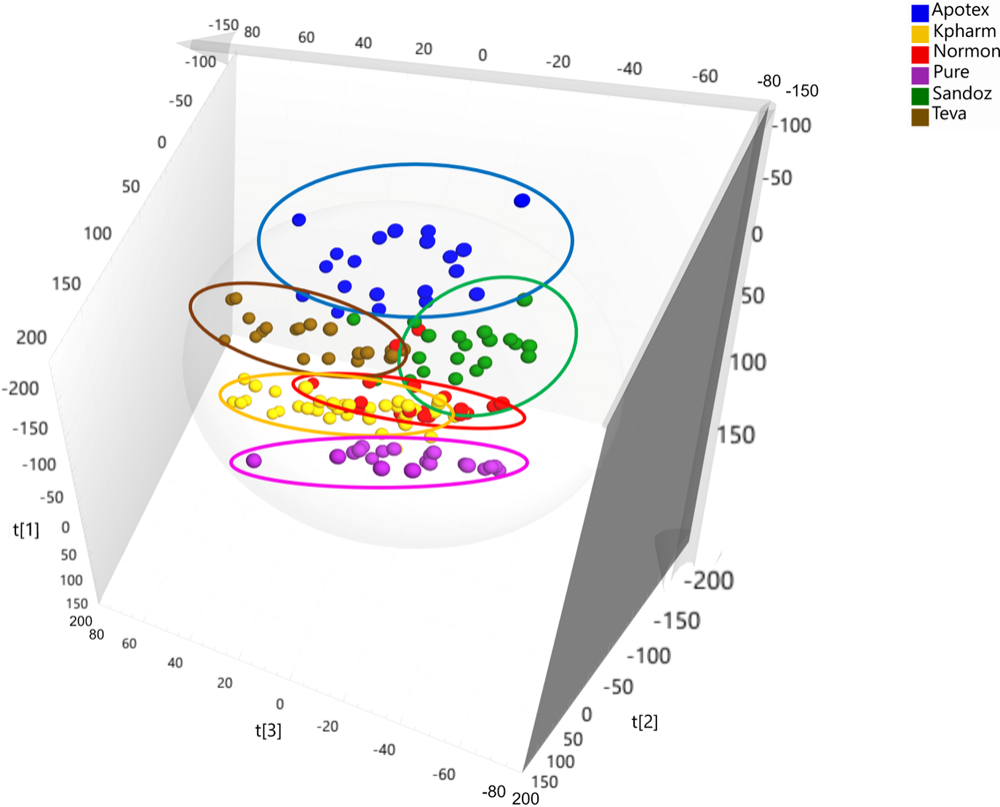
PCA score scatter 3D plot for all the spectra samples except (left) both oral ERNSA and granulated forms; (right) both oral ERNSA and granulated, and pure, forms. The samples are colored by Brand.

Afterward, in order to focus the analysis on the tablets (inside the bubble), the students were asked to exclude the IR spectra from the Algidol powder and the Apiretal oral solution from the subsequent PCA analysis (Figure 6). In the resulting plot, the tablets spectra were colored based on their brand. It should be noticed that all brands were visually distinguishable. The scores belonging to different samples (independently colored) were not mixed-up within each other but sequentially distributed along the graph into various clusters that are rather easy to differentiate. However, the boundaries of some clusters overlapped each other as can be observed for the *Normon* and *Kpharm* samples, which entangled enough to confuse their brands.

**Figure 7:**
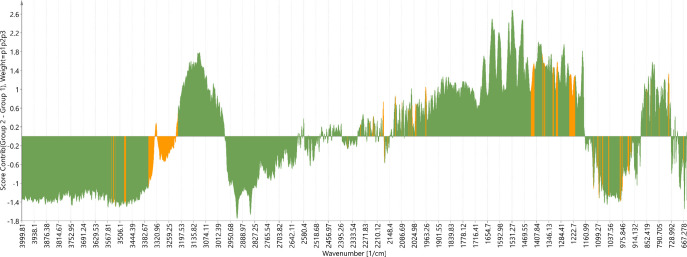
Contribution (loading modified) plots for differentiating the Apotex (group 2: positive-located spectra) from the Sandoz brand (group 1: negative-located spectra). The most important bands and shoulders were automatically colored in orange because they are remarkable since they were outside the three standard deviation range.

Besides the PCA scores visualization, the students were invited to visualize the loadings responsible for that PCA distribution. It is chemically useful to find out which IR bands are responsible for the differentiations observed in the scores. For instance, when trying to understand which bands separated the Sandoz brand from the Apotex brand, the students observed in the contribution plot (Figure 7), that the positive-located bands belonged to Apotex while the negative-located bands belonged to the Sandoz brand. In this case, the most important bands and shoulders for differentiating Apotex from Sandoz were automatically colored in orange on the positive-located bands. These bands are remarkable because they were outside the three standard deviation range. Subsequently, the next most important bands for the differentiation were represented by the highest intensity peaks in that plot. In the opposite side of the plot, the most important bands and shoulders for the differentiation of the Sandoz brand from Apotex were colored in orange on the negative-located bands, meaning that they were outside the three standard deviation range (Figure 7). These bands were mostly due to O–H/N–H stretching vibrations (3600–3200 cm^–1^), aromatic C–H (C(sp^2^)–H) stretching (3100–3030 cm^–1^), C–H (C(sp^3^)–H) stretching (3000–2800 cm^–1^), N–H amide II (1630–1500 cm^–1^), O–H ip (in-plane) bending (1450–1200 cm^–1^), and aromatic C–H ip bending (1250–950 cm^–1^). Thus, the Apotex and Sandoz brands had small but statistically significant differences in the components ratio comprising these chemical bonds.

#### iii Classification

In chemometrics, classification involves examining whether a sample belongs to a particular predefined class/group. Once the exploration, visualization, and differentiation steps are finished, other more powerful yet more complicated techniques can be used to classify the samples visualized by PCA. Examples of those techniques are projection to latent structures or orthogonal partial least squares (PLS),^29−31^ orthogonal projection to latent structures or orthogonal partial least squares discriminant analysis (OPLS-DA),^12^ and others.^32−35^ These methods could be used to try discriminating the entangled Normon and Kpharm samples. In general, such methods may, for example, return plots or misclassification/confusion matrices that indicate how many samples are properly classified or not according to certain criteria (Table 3). For the studied drugs, all brands were properly classified using OPLS-DA, including the entangled Normon and Kpharm samples (Table 3). The classification methods are only suggested if the students are in advanced degree courses (or even at postgraduate level like master projects). This part of the lesson was demonstrated by the teacher but not performed by the students. However, it is interesting to collect it in this work as a guide for more advanced chemometrics courses and to see the global and sequential view of what chemometrics can contribute to the study of IR spectroscopy.

**Table 3:** Summary of How Well a Particular OPLS-DA Classification Model Classified the Samples into the Known Classes

			Distribution of Classification Results by Brand Name					
Sample Source	Members	Correct, %	Apotex	Kpharm	Normon	Pure	Sandoz	Teva
Apotex	20	90.0	18^a^	0	0	0	1^b^	1^b^
Kpharm	40	97.5	0	39^a^	1^b^	0	0	0
Normon	20	100.0	0	0	20^a^	0	0	0
Pure	20	100.0	0	0	0	20^a^	0	0
Sandoz	20	100.0	0	0	0	0	20^a^	0
Teva	20	100.0	0	0	0	0	0	20^a^
Total	140	97.9						

a Proportion of correctly classified samples in the data set.

b Proportion of incorrectly classified samples.

## Conclusions

ATR-FTIR is an environmentally friendly, solvent-free, fast, nondestructive technique for the identification of chemical substances. In addition, the identification capability of IR spectroscopy makes ATR-FTIR spectroscopy a very useful and widely used technique in many fields, such as chemistry, pharmacy, forensics, and food analysis, among others experimental sciences. A practical training in these methodologies is essential for undergraduate students, including instrumental optimization, vibrational interpretation of infrared bands, chemical identification of major components, and chemometric discrimination of similar samples.

The different lessons previously described contribute to a significant learning in the ATR-FTIR spectroscopy field. In this case, the application of this technique to face an interesting problem such as the analysis of drugs makes this study more effective for students.

Regarding the instrumental conditions, the students learned that the spectral resolution strongly influences the sharpness (definition) of the IR spectrum, whereas the number of scans influences the spectral noise. They learned that the optimum parameter values are usually selected by considering a balance between the sharpness and the signal-to-noise ratio.

Infrared absorption bands are due to fundamental molecular vibrations (stretching and bending modes). Hence, every IR spectrometer user should have a basic knowledge about the correlation between IR bands and the chemical groups/bonds in the molecule she/he is trying to identify. In this case, the interpretation of the experimental IR bands against the chemical bonds of the acetaminophen (paracetamol) and ascorbic acid (vitamin C) molecules was deeply accomplished and discussed. The study of these two molecules, which involves the presence of different chemical groups such as N–H (amide), C(sp^2^)–H (aromatic), and C=O and C–N–C (amide) in acetaminophen, versus C=O and C–O–C (cyclic ester) in ascorbic acid, provides the students with wide knowledge about the different type of bonds present in a wide range of chemical compounds.

The FTIR spectrum is like a fingerprint for each molecule, in such a way that an unknown spectrum might be compared with spectral libraries in order to identify it. The students learned that this process can be automatically performed by the infrared spectroscopic software. The software provides a list of more similar candidates, after calculating the statistical matching (Pearson coefficient) between the unknown spectrum and all the spectra along a particular local or online library. In this study, acetaminophen was identified in all drugs except in the Apiretal drug.

Finally, a chemometric multiple sample comparison was presented to the students to find out the coarse differences among the spectra of several commercial drugs containing the same API. However, a more powerful yet rather simple and graphical chemometric tool like PCA was presented to the students to visualize most of the samples. Consequently, chemometric tools are useful to discriminate samples having slight differences in their spectra (caused by their varying composition), which might not be detectable to the naked eye.

## Supporting Information Available

Background fundamental theoretical explanation about vibrational spectroscopy and chemometrics that undergraduate students should know before tackling these laboratory experiments (PDF)
