# Supplementary material for: Introducing ATR-FTIR Spectroscopy through Analysis of Acetaminophen Drugs: Practical Lessons for Interdisciplinary and Progressive Learning for Undergraduate Students
Source: J Chem Educ. 2021 Jul 12;98(8):2675–86. doi: 10.1021/acs.jchemed.0c01231 (PMC8908246; doi:10.1021/acs.jchemed.0c01231)
Supplement: Supplementary file 1 — ed0c01231_si_001.pdf [file ed0c01231_si_001.pdf]

# Introducing ATR-FTIR Spectroscopy through Analysis of Acetaminophen Drugs: Practical Lessons for Interdisciplinary and Progressive Learning for Undergraduate Students

## SUPPLEMENTARY MATERIAL

### Useful fundamental concepts the student should know/remember before tackling the laboratory practice

Matter is composed of atoms. These atoms can be found free (*i.e.* elements) or bonded (*i.e.* forming chemical compounds). In the latter case, depending on the type of bond, compounds can be molecules (covalent bond), ionic compounds (ionic bond), or metals / alloys (metal bond).[1] The field that studies the interaction between radiation and atoms, with the aim of obtaining atomic information, is called atomic spectroscopy.[2] The field that studies the interaction between radiation and molecules, with the aim of obtaining molecular information, is called vibrational spectroscopy,[3] because radiation makes molecules to vibrate.

Molecules specifically absorb the radiation whose energy is the same as that required to produce the molecular vibrations. In the case of fundamental molecular vibrations, this frequency belongs to medium-infrared (MIR) radiation. This way, molecular vibrations provide characteristic absorption bands within IR range. However, no every molecular vibration is IR active. The selection rules of IR spectroscopy establish that only those molecular vibrations that produce a change in the dipole moment (μ) of the molecule are IR active.[2-4] According to the Planck-Einstein equation ($\boldsymbol{E} = h{*}\nu = h{*}c/\boldsymbol{\lambda} = h{*}c{*}\tilde{\nu}$), wavelength is inversely proportional to the radiation energy. Instead of wavelength (λ), in MIR it is common to use wavenumber ($\tilde{\nu}$), which is the inverse of the wavelength. Thus, wavenumber is directly proportional to the energy.[1-4] Particularly, MIR range covers the region from 4000 to 400 $cm^{-1}$ (wavenumbers).

The energy absorbed in a molecular vibration depends on three factors: the type of vibration, the atoms that are involved and the vicinity of surrounding atoms. The first two factors mainly determine the energy of the vibration, and therefore, the main location of its IR absorption band along the IR spectrum, whereas the latter factor provides small shifts in the frequency (wavenumber) of the vibration, that are certainly useful to selectively identify each molecule. Because of these reasons, IR spectrum is characteristic for each molecule, in such a way that the IR pattern is like a molecular fingerprint, useful to unequivocally identify every molecule.[2]

The maximum number of fundamental molecular vibrations of a certain molecule containing N atoms is theoretically calculated as 3N-6 (for non-linear molecules) or 3N-5 (for linear molecules).[3,4] These values result from the combination of the three degrees of freedom available for every atom (3N) and the removal of those translational/rotational non-detectable modes (-6 or -5). These molecular vibrations are mainly classified into stretching and bending vibrations depending on whether the bond-distance changes during the vibration or keeps constant. Those vibrations during which the bond distance changes are named stretching vibrations, while the vibrations during which the bond distance keeps constant, but the angle of bonds changes, are named bending vibrations.[3] Stretching vibrations are further sub-classified into symmetric and antisymmetric stretching vibrations (depending on whether atoms move specularly or not). Similarly, bending vibrations are also sub-classified into in-plane (ip) and out-of-plane (oop) vibrations, as schematized in Figure S1. In plane vibrations are differentiated into scissoring (atoms move towards and away from each other in-plane) or rocking (atoms move pendularly in-plane). Oop vibrations are subclassified into wagging (atoms move back and forth out-of-plane) or twisting (one atom moves forward while the other one moves backwards both out-of-plane).[6] Stretching vibrations normally require more energy than bending vibrations. Therefore, the bands corresponding to stretching vibrations are located at higher wavenumbers than the respective bending vibrational bands. For instance, the band due to -OH stretching vibration allocates within 3650-3000 $cm^{-1}$, whereas the band due to -OH ip bending allocates within the range 1400-1200 $cm^{-1}$. In addition, antisymmetric stretching requires more energy, that supposes higher wavenumber, than symmetric stretching, (*e.g.,* -$NO_2$ antisymmetric (1650-1500 $cm^{-1}$) vs -$NO_2$ symmetric (1390-1260 $cm^{-1}$) stretching vibrations).[3] IR bands can be also classified as strong (s), medium (m), or weak (w), depending

on the relative transmission intensity of radiation in the IR spectrum. A strong band indicates high % transmission in relation to the light emitted and covers most of the *y*-axis, a medium band falls to about half of the *y*-axis, and a weak band indicates low % IR transmission and falls to about one third or less of the *y*-axis. The intensity of the IR bands mainly depends on the magnitude of the dipole moment associated with the chemical bonds.

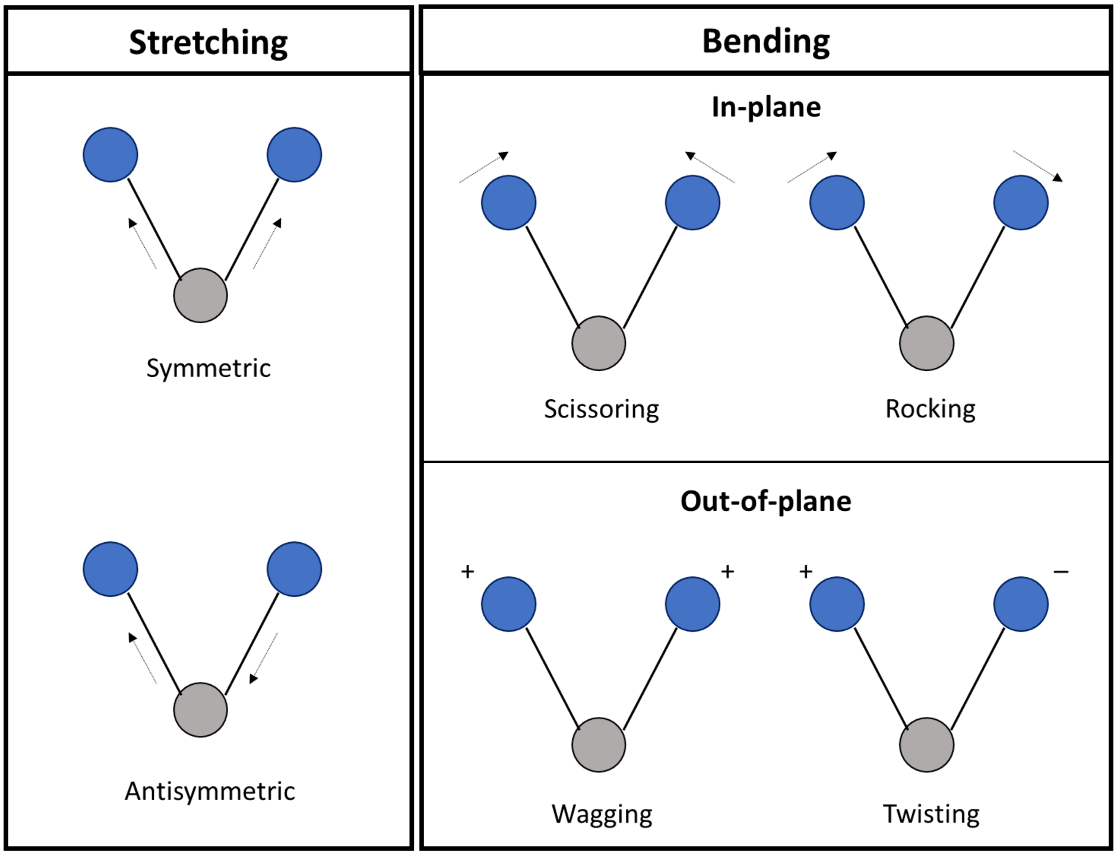


***Figure S1***. *Scheme of fundamental stretching and bending molecular vibrations.*

The type of atoms involved in the vibration also influence to a large extent the energy of the vibration. In general, the smaller and closer the atoms are, the higher energy they need to vibrate, and hence the higher the wavenumber is. This is in accordance with the Hooke's law for harmonic oscillators in which the required energy to move two linked objects, in this case two bonded atoms, is inversely proportional to the distance of both objects. In addition, the more strongly bonded the atoms are, the higher the wavenumber of vibration is. Thereby, the stiffness/strength of the chemical bond, *i.e.,* whether it is a single, double or triple bond, directly correlates with the wavenumber, whereas the bond distance is

inversely correlated.[3] As an example, C-H stretching vibrations (3100-2840 $cm^{-1}$) require more energy than C-O stretching vibrations (1300-970 $cm^{-1}$), as expected for smaller H atom because of a much shorter bond distance. Similarly, C=O stretching (1800-1630 $cm^{-1}$) requires more energy than C-O stretching (1300-970 $cm^{-1}$), as expected for stronger double bond.[3]

Besides comprehending previous basic IR vibrational concepts, students are encouraged to review some fundamental IR instrumental concepts. In this respect, students need to know that the main IR instrumental/methodological advance involves the use of interferometry and mathematical Fourier transformation, which occurred in the 1970s with the arrival of computers to spectroscopy.[2] Interferometry was largely developed by Michelson in the 1880s. However, the complex interferograms could not be interpreted until computers rapidly calculated the mathematical Fourier transformation. Interferograms represent the variation of the radiation energy as a function of the time lapse between two beams travelling through different optical paths inside an interferometer (one of them to the sample).[2-3] Applying Fourier transformation, interferograms are converted into IR spectra, in which absorption intensity is displayed as a function of wavenumber ($cm^{-1}$). This way, the interpretation and comparison of IR absorption bands is much more straightforward and faster.

In addition, students need to know that, regarding the physical type of interaction between matter and IR radiation, FTIR spectroscopic techniques are further sub-classified into transmission and reflection modes.[2-4]

Before 1960, transmission was the unique mode used in IR spectroscopy due to its simpler instrumental design. In the transmission mode, IR radiation simply passes through the sample, reaches the detector and the fraction of the incident radiation that has been absorbed by the sample is determined. Maximum signal reaches the detector since incident and transmitted light travel in one unique straight direction. The main disadvantage of transmission IR spectroscopy lies on the fact that a sample treatment is usually required because a thin layer of substance is essential to enable that a significant amount of transmitted radiation reaches the detector. Otherwise, the substance might totally absorb IR radiation and no transmitted radiation would be detected at all. This sample treatment is different depending on whether the substance is liquid or solid. If the substance is liquid, a small volume is spread between two potassium bromide pellets. If the substance is solid, there are two methods to

prepare the sample: making a diluted solution or preparing a thin layer of the substance. To prepare a solution, the solvent is recommended to provide no IR intense overlapping bands, such as bromoform or nujol, and the solution is spread between the pellets, as mentioned before. To prepare a thin solid layer, the most common procedure involves powdering the sample in a mortar, mixing a spatula tip of powdered sample with potassium bromide powder (non-IR active) and pressing the mixture to form a very thin pellet.[2-4] In any case, those samples cannot be reused for further experiments.

Attenuated total reflection (ATR), method developed in the 1960s, is based on the refractive index of substances. When two substances of high and low refractive index are placed in contact, incident light travelling through the material of higher refractive index gets almost totally reflected when it reaches the interface between both materials because of the low refraction index of the second material. Particularly, the reflected light penetrates a short distance, of the order of the wavelength, into the substance of lower refractive index, in such a way that reflected radiation is affected by the interaction with that substance.[2-4] ATR accessories incorporate a flat ATR plate of a high-refractive-index material such as germanium, or diamond, on which the sample might be placed and pressed using the pressing device that is usually coupled to IR systems. Since radiation shortly penetrates into the sample, the pressing of the sample against the ATR plate is crucial to record the IR spectrum. Hence, contrarily to transmission mode, sample can be directly analysed by ATR with no treatment, which is a significant advantage.[3]

In either transmission or reflection modes, the Fourier transformation of the interferogram provides an IR spectrum. However, depending on the type of radiation that is being recorded, the parameter that is measured is different: transmittance in transmission mode *versus* reflectance in reflection mode. Transmittance is often mathematically converted into absorbance ($A = -\log T$) when displaying the IR spectrum. Similarly, reflectance is often converted into $-\log R$. The reflectance spectrum of a substance is not identical to its transmittance spectrum. Particularly, the relative intensity of certain bands may change and small shifts in the wavenumber of certain bands might occur.[2-4] Positively, they are slight differences, which do not usually affect the identification of substances.

At this point, a related topic to also learn in this course is the chemometric analysis applied to IR spectroscopy. Chemometrics is defined by the IUPAC as "the application of statistics to the analysis of

chemical data (from organic, analytical or medicinal chemistry) and to the design of chemical experiments and simulations".[5,6] Chemometrics are often applied without a significant learning by students. In various fields of science, chemistry and statistics are taught simultaneously or in progressive courses. The collaboration among different subjects can increase a significant learning. In essence, regarding this work, the treated data has a chemical meaning because the IR spectra represents characteristic chemical fingerprints of the analysed compounds. It should be noted that the IR spectra are multidimensional data because they are a continuous average of the compounds' IR transmittance/reflectance as a function of the frequency. In many areas of research more than one IR spectrum is often collected for each sample. Therefore, the use of a bunch of IR spectra for studying chemometrics can be an opportunity to make it relevant whilst reaching a significant learning in both subjects. There are a few ideas that are worth mentioning: a) the data treated using chemometrics are normally multivariate *per se*; b) although sometimes the mathematical and statistical methods used in chemometric applications are the same as those used in theoretical chemistry, chemometrics does not imply theoretical calculations but the extraction of the useful chemical information stored in the measured data; and c) chemometrics is broadly used outside chemistry, in fields like biology, metabolomics, engineering, forensic science, cultural studies, etc. Hence, although there are quite many chemometric methods (Figure S2), the proposed course includes some basic chemometric concepts and methods for comparing the IR spectra and for exploring the underlying characteristics of the analysed samples, which come from larger data populations.

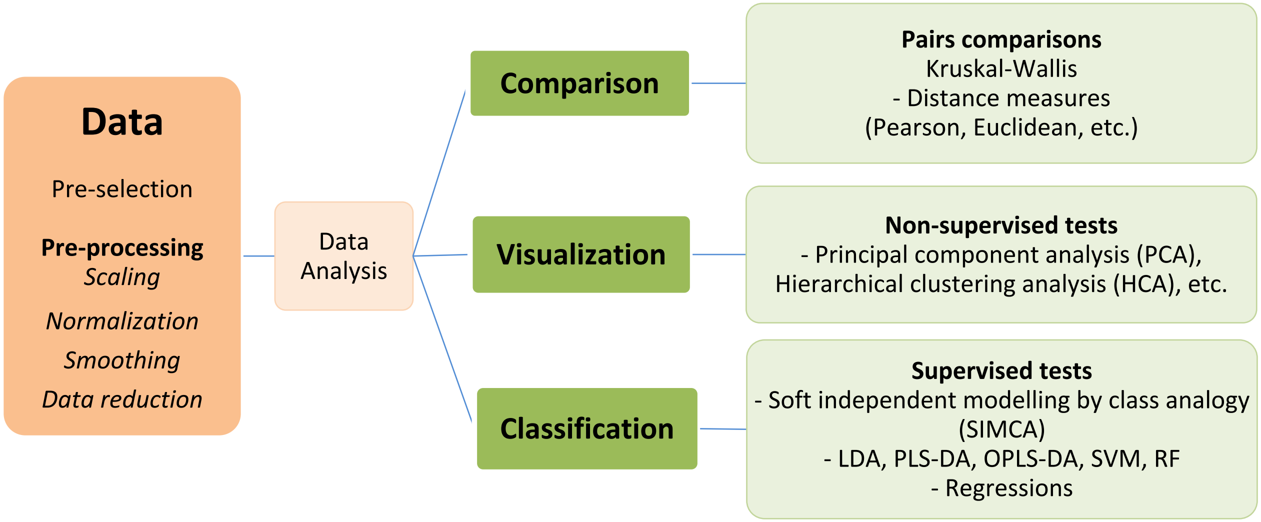


***Figure S2***. *Scheme commonly followed when applying chemometrics to FTIR data.*

Some of those chemometric methods comprise the highly used principal components analysis (PCA),[7-9] or other more advance chemometric techniques.[10-13] PCA is a data reduction and exploration technique that helps visualizing how statistically similar or different the samples (spectra) are, while obtaining an overview of their main sources of variability.[7-11] This is done by plotting the scores (which represent the samples) and loadings (which represent the variables) in order to explain the differences found among the sample classes (samples' groups organised row-wise).

## REFERENCES


1. Buthelezi, T.; Dingrando, L.; Hainen, N.; Wistrom, C.; Zike, D. Chemistry. Matter and change, Glencoe/Mc Graw Hill, **2008**.
2. Chalmers J.; Griffiths P. Handbook of vibrational spectroscopy, Wiley, **2002**.
3. Zapata F. Vibrational spectroscopy for the characterization of explosive residues and body fluids, PhD Thesis, University of Alcalá, **2018**.
4. Theophanides, T. Infrared spectroscopy – Materials Science, engineering and Technology, In Tech, **2012**.

5. McNaught, A. D. Wilkinson. A. IUPAC. Compendium of Chemical Terminology, 2nd ed. (the "Gold Book"). IUPAC Gold Book. S. J. Chalk. Oxford, Blackwell Scientific Publications. **1997**.
6. Van De Waterbeemd, H.; Carter, R. E.; Grassy, G.; Kubinyi, H.; Martin, Y. C.; Tute M. S.; Willett P. "Glossary of terms used in computational drug design (IUPAC Recommendations 1997)." Pure and Applied Chemistry **1997**, 69(5), 1137.
7. Massart, D. L.; Vandeginste, B. G. M.; Buydens, L. C.; De Jong, S.; Lewi, P. J.; Smeyers-Verbeke J. Principal Components. Data Handling in Science and Technology. Handbook of Chemonietrics and Qualimetrics: Part A. Vandeginste. B. G. M., R. S.C. The Netherlands, Elsevier. **1997**, 20A, 519-556.
8. Massart, D. L.; Vandeginste, B. G. M.; Deming, S. N.; Michotte Y.; Kaufman L. Principal Components and Factor Analysis. Data Handling in Science and Technology. Chernornetrics: a textbook. Vandeginste B. G. M.; and Kaufman, L. The Netherlands, Elsevier. **2003**, 2, 339-370.
9. Eriksson, L.; Byrne, T.; Johansson, E.; Trygg, J.; Vikström C. PCA. Multi- and Megavariate Data Analysis Part I: Basic Principles and Applications, Umetrics. **2006**, AB, 39-62.
10. Eriksson, L.; Byrne, T.; Johansson, E.; Trygg, J.; Vikström C. Classification and discrimination. Multi- and Megavariate Data Analysis Part I: Basic Principles and Applications, Umetrics. **2006**, AB, 171-194.
11. Miller, J. N.; Miller, J. C. Multivariate analysis. Statistics and Chemometrics for Analytical Chemistry. London, Pearson Education Limited. **2010**, 221-250.
12. Massart, D. L.; Vandeginste, B. G. M.; Buydens, L. C.; De Jong, S.; Lewi, P. J.; Smeyers-Verbeke J. Supervised Pattern Recognition. Data Handling in Science and Technology. Handbook of Chemonietrics and Qualimetrics: Part B. B. G. M. Vandeginste. R. S.C. The Netherlands, Elsevier. **1998**, 20B, 207-242.
13. Heise, H. M.; Winzen R. Chemometrics in Near-Infrared Spectroscopy. Near-In frared Spectroscopy: Principles, Instruments, Applications. Siesler, H. W.; Ozaki, Y.; Kawata S.; and Heise, H. M. Weinheim, Wiley-VCH. **2002**, 125-162.
